# Structure and Properties of PSf Hollow Fiber Membranes with Different Molecular Weight Hyperbranched Polyester Using Pentaerythritol as Core

**DOI:** 10.3390/polym12020383

**Published:** 2020-02-08

**Authors:** Min Liu, Long-Bao Zhao, Li-Yun Yu, Yong-Ming Wei, Zhen-Liang Xu

**Affiliations:** 1 Key Laboratory for Ultrafine Materials of Ministry of Education, Shanghai Key Laboratory of Advanced Polymeric Materials, School of Materials Science and Engineering, East China University of Science and Technology (ECUST), 130 Meilong Road, Shanghai 200237, China; 2 State Key Laboratory of Chemical Engineering, Membrane Science and Engineering R&D Lab, Chemical Engineering Research Center, ECUST, 130 Meilong Road, Shanghai 200237, China; killt@live.cn (L.-B.Z.); ymwei@ecust.edu.cn (Y.-M.W.); 3 Danish Polymer Center, Department of Chemical and Biochemical Engineering, Technical University of Denmark, Building 227, 2800 Kgs. Lyngby, Denmark; lyyu@kt.dtu.dk

**Keywords:** hyperbranched polyester, molecular weight, polysulfone, hollow fiber membranes

## Abstract

A homologous series of hyperbranched polyesters (HBPEs) was successfully synthesized via an esterification reaction of 2,2-bis(methylol)propionic acid (bis-MPA) with pentaerythritol. The molecular weights of the HBPEs were 2160, 2660, 4150 and 5840 g/mol, respectively. These HBPEs were used as additives to prepare polysulfone (PSf) hollow fiber membranes via non-solvent induced phase separation. The characteristic behaviors of the casting solution were investigated, as well as the morphologies, hydrophilicity and mechanical properties of the PSf membranes. The results showed that the initial viscosities of the casting solutions were increased, and the shear-thinning phenomenon became increasingly obvious. The demixing rate first increased and then decreased when increasing the HBPE molecular weight, and the turning point was 2660 g/mol. The PSf hollow fiber membranes with different molecular weights of HBPEs had a co-existing morphology of double finger-like and sponge-like structures. The starting pure water contact angle decreased obviously, and the mechanical properties improved.

## 1. Introduction

Due to its low cost, superior membrane-forming ability and good mechanical and anti-compaction properties, polysulfone (PSf) is widely used to prepare ultrafiltration (UF) membranes [1,2,3]. However, the hydrophobic nature of PSf leads to the low permeability and high fouling of the PSf membranes, which reduce the membranes’ life and limit their application [4,5]. Therefore, the modification of PSf membranes is becoming increasingly important.

To improve surface hydrophobicity and membrane permeability, many efforts have been devoted to membrane modification, including chemical modification [6,7,8], irradiation modification [9,10], blending modification [11,12,13,14,15,16] and so on. Compared with other methods, blending modification is an effective and convenient approach due to its facile operation and excellent modification efficiency. Liu et al. [12] used dicyclohexylbenzene amide (TMB-5) as a nucleating agent to prepare polyvinylidene fluoride (PVDF) microporous membranes via thermally induced phase separation (TIPS) and investigated the effects of TMB-5 on the PVDF membranes. With the addition of TMB-5, the structures of the membranes were interconnected and bi-continuous. Yuan et al. [13] investigated the effects of perfluorosulfonic acid (PFSA) on PVDF hollow fiber membranes. With the addition of PFSA, the morphology of the membrane cross-section was altered, and finger-like macrovoids developed from inside the skin layer of the nascent membrane. With an increase in the PFSA content, the membranes showed improved wetting properties. Xu et al. [16] studied the effects of poly(ethyleneglycol) (PEG) with various molecular weights (${\bar{M}}_{w}$ = 200, 600, 2000, 6000 and 10,000 Da) on the performance of polyethersulfone (PES) hollow fiber ultrafiltration membranes. They reported that not only the additive content but also the additive molecular weight significantly determined the performance of the membranes. When the molecular weight of PEG increased from 200 to 10,000 g/mol, the membrane structures were converted from a double-layer finger-like structure to microvoids in the form of spheres or ellipsoids, and the pure water permeation fluxes increased from 22.0 to 64.0 L/m^2^h, but the mechanical properties worsened.

Hyperbranched polyesters (HBPEs) based on 2,2-bis(methylol)propionic acid (bis-MPA) have been a research focus in recent years due to their high functionality, globular structure, low solution, melt viscosity, good thermal stability and high solubility [17,18,19]. Currently, few studies have reported using HBPEs as additives to prepare hollow fiber membranes, but Zhao et al. [20,21,22] investigated the effect of hyperbranched polyglycerol (HPG) and its derivatives on the morphology and properties of polyvinylidene fluoride porous membranes. Compared with membranes modified by linear PEG as additives, HPG not only acted as a pore-forming agent but also as a hydrophilic modifier. In our previous work [23], the effects of HBPE content on the structure and properties of PSf hollow fiber membranes were discussed. Our research suggested that the prepared membranes had good hydrophilicity and exhibited good permeability. Sun et al. [24] prepared a novel composite membrane of cross-linked poly(vinyl alcohol) (PVA)/HBPEs and investigated the effects of the HBPE content on the performance of the PVA/HBPE membranes. Their research suggested that the prepared membranes had good hydrophilicity and exhibited good permeability.

In this work, we synthesized a series of HBPEs with different molecular weights using pentaerythritol (PER) as a core and used it as an additive to prepare PSf hollow fiber membranes. By using HBPEs with different molecular weights, we focused on its effects on the morphology, hydrophilicity and mechanical properties of PSf hollow fiber membranes.

## 2. Materials and Methods 

### 2.1. Materials

PSf was purchased from Sino Polymer Co. Ltd. of East China University of Science and Technology (ECUST, Shanghai, China). The polymer was used as the membrane material and dried at 333 K prior to processing. Acetone, ether, *N,N*-dimethylacetamide (DMAc) and (poly)ethyleneglycol (average molecular weight of 400), were obtained from Shanghai Chemical Agent Co. Ltd. (China). p-toluenesulfonic acid (p-TSA) and pentaerythritol (PER) were purchased from Shanghai Chemical Agent Co. Ltd. (China). 2,2-bis(methylol)propionic acid was purchased from Tokyo Chemical Industry Co. Ltd. (Japan). Dextrans (${\bar{M}}_{w}$ = 40, 70, 100, 500 and 2000 kDa, respectively, analytical grade, Sigma-Aldrich Co., Ltd., St. Louis, MI, USA) were used to characterize the performance of PSf hollow fiber membranes. 

### 2.2. Synthesis and Characterization of HBPEs

The HBPEs were synthesized on the basis of the method reported in the literature [19]. Measured amounts of PER, bis-MPA and p-TSA were placed into a four-neck glass flask. p-TSA was the acid catalyst of the esterification reaction, and bulk polymerization was performed at 413K. The subsequent experiment process is the same as previously reported in our previous work [23]. Four HBPEs (SP-1, SP-2, SP-3 and SP-4) with different molecular weights were synthesized by changing the ratio between PER and bis-MPA. The chemical structures of the synthesized HBPEs were characterized by a Fourier-transform infrared spectroscopy (FTIR, Nicolet 6700, Thermo Electron Scientific Instruments Corporation, Waltham, MA, USA). The average molecular weight of the HBPEs was identified by gel permeation chromatography (GPC, Waters 1525, Milford, MA, USA) using polystyrene for calibration. 

### 2.3. Preparation of PSf Hollow Fiber Membranes

The compositions of the casting solutions are listed in Table 1. Homogeneous casting solutions were acquired by stirring the solution for 24 h at 298 K and degassing to remove air bubbles at room temperature and constant pressure. 

Subsequently, the PSf membranes were spun via a non-solvent induced phase separation process at 298K. During the spinning process, pure water was used as the bore fluid solution and external coagulation baths. The dope and the bore flow rate were invariable, and the details of the spinning process were described in our previous works [16,25]. As is well known to all, the drying or pre-treatment procedure has an important influence on the membrane structure and separation properties. Albo et al. [26,27,28] reported in detail the effect of the pre-treatment procedure on the morphology of the membrane. In the present study, the residual solvent in these prepared membranes were extracted with pure water for 3 days. Then, these hollow fiber membranes were immersed in 20 wt % glycerol aqueous solution for 3 days to prevent the collapse of the membrane structure and then dried for at least 48 h at room temperature to obtain dry membranes before testing. 

### 2.4. Characterization of Casting Solutions

#### 2.4.1. Viscosity Measurements

The viscosities of the PSf/HBPE/PEG400/DMAc casting solutions were measured with a digital viscometer (DV-II+PRO, Brookfield, Middleboro, MA, USA) at 298 K. The recorded data were the curve of shear viscosity to the shear rate.

#### 2.4.2. Light Transmittance Measurement 

To determine the demixing rate of the casting solution, light transmittance measurement experiments were presented. The experiment was reported by Liu et al. [29]. The light transmittance intensity through the PSf membrane was recorded as a function of immersion time.

### 2.5. Membrane Characterization

#### 2.5.1. Morphology

The PSf morphologies of the fabricated membranes were observed by scanning electron microscopy (SEM, JEOL Model JSM-6380 LV, Tokyo, Japan). The PSf membranes were fractured in liquid nitrogen and sputtered with gold under a vacuum.

#### 2.5.2. Hydrophilicity and Porosity

The hydrophilicity of the prepared membranes was measured by the dynamic pure water contact angle (θ) of the PSf membrane’s outer surfaces. A contact angle meter (JC2000A, Zhongchen Digital Equipment Co., Ltd., Shanghai, China) was used at room temperature. After a water droplet (0.2 μL) dispersed on the membrane’s outer surface, a camera captured images at 10 frames/s, and the θ was measured via the specific software from these images. The reported data were measured five times for each sample and averaged.

Membrane porosity (ε, %) of the fabricated membranes was determined by the dry–wet weight method. The detailed procedure was reported by Zhao et al. [23]. The porosity was calculated as the following formula, Equation (1):

(1) $$\varepsilon =\frac{\left(m_{W}-m_{D}\right)/\rho_{H_{2}O}}{\left(m_{W}-m_{D}\right)/\rho_{H_{2}O}+m_{D}/\rho_{P}}\times 100\%$$

where *m_D_* is the weight of the dry membrane (g), *m_W_* is the weight of the wet membrane (g), *ρ_P_* is the PSf density (g/cm^3^) and *ρ_H2O_* is the pure water density (g/cm^3^).

#### 2.5.3. Permeation Property

The pure water permeation flux (*J_w_*) and rejection rate (*R*) were measured using a self-prepared apparatus [30]. Before testing, the pre-assembly modules were immersed into pure water to remove the residual glycerol. Pure water and dextran aqueous solutions (500 ppm) were used as the feed solutions. All the testing was carried out at room temperature. Before the *J_w_* and the *R* of dextran aqueous solution were measured, the pre-treatment modules were pre-pressured at 0.1 MPa for 1 h. The dextran contents of the feed and permeate solutions were detected by the total organic carbon instrument (TOC, Shimadzu TOC-VCPH, Tokyo, Japan). The *J_w_* and the *R* were determined by Equations (2) and (3), respectively

(2) $$J_{w}=\frac{Q}{t\times A}$$

(3) $$R=\left(1-\frac{C_{P}}{C_{F}}\right)\times 100\%$$

where *J_w_* is the pure water permeation flux (L/m^2^ h), *A* is the effective area of the membrane (m^2^), *Q* is the volume of the permeate pure water (L) and *t* is the permeation time. *R* is the rejection rate of the dextran, and *C_P_* and *C_F_* are the dextran concentrations of the permeate and feed solution, respectively.

#### 2.5.4. Pore Size Distribution and Molecular Weight Cut-off (MWCO)

The pore size distribution and the molecular weight cut-off were obtained by a series of dextrans rejection experiments, which has been used by many researchers [13,31,32,33,34]. The log-normal distribution function was defined as follows:

(4) $$f(d_{p})=\frac{dR\left(d_{p}\right)}{dd_{p}}=\frac{1}{d_{p}\ln\varepsilon_{p}\sqrt{2\pi }}\exp\left[-\frac{{\left(\ln d_{p}-\ln\bar{d_{p}}\right)}^{2}}{2{\left(\ln\varepsilon_{p}\right)}^{2}}\right]$$

where ${\bar{d}}_{p}$ is the geometric mean diameter and *ε_p_* is the geometric standard deviation. The ultrafiltration experiment was used to determine the two parameters and MWCO using dextran (${\bar{M}}_{w}$ = 40, 70, 100, 500 and 2000 kDa, respectively) solutions. 

#### 2.5.5. Mechanical Properties

The mechanical properties (breaking strength, elongation at break and Young’s modulus) of the membranes were measured by a material testing machine (QJ210A, Shanghai Qingji Instrumentation Science & Technology Co., LTD, China). The loading velocity was 50 mm/min. Each sample was measured five times and then averaged. 

## 3. Results and Discussion

### 3.1. Characterization of HBPEs

**Figure 1:**
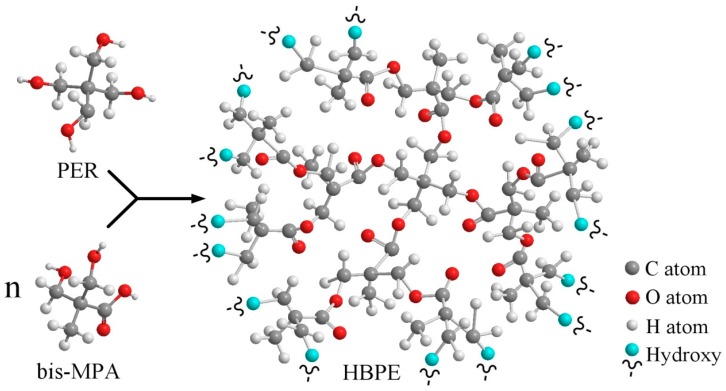
Schematic chemical structure of the HBPEs formed by esterification reaction of pentaerythritol (PER) with bis(methylol)propionic acid (bis-MPA).

**Figure 2:**
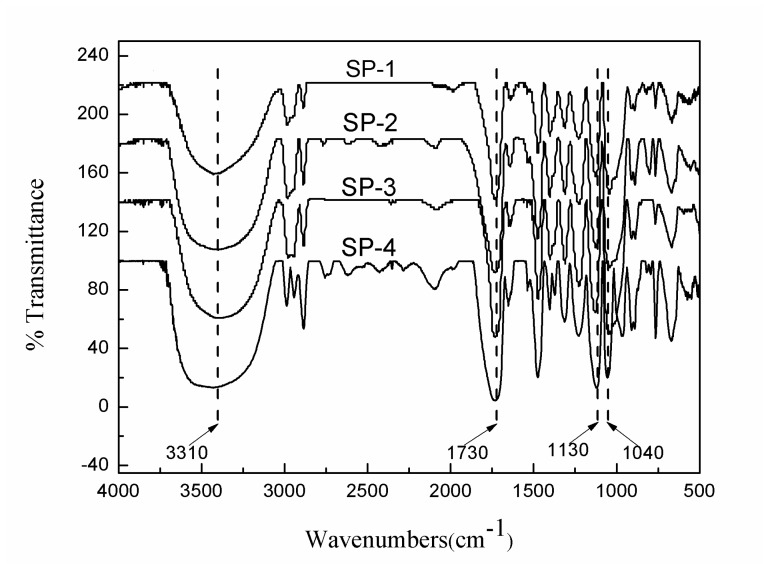
The Fourier-transform infrared spectroscopy (FTIR) spectra of the prepared HBPEs.

Bis-MPA with two hydroxyl groups and one carboxyl group is usually used as an AB_2_ (A = –COOH and b = –OH) monomer to prepare HBPEs. The chemical structure of the HBPEs based on PER and bis-MPA is shown in Figure 1. Four types of HBPEs with various molecular weights were synthesized by changing the ratio between PER and bis-MPA. As shown by the FTIR spectra (Figure 2), the absorption peaks of the –OH typical stretching vibration at 3310 cm^−1^ could be observed for all of the HBPEs. This indicates that there are many hydroxyl groups in the prepared HBPE molecules. Moreover, the characteristic stretching vibrations of carbonyl groups at 1730 cm^−1^, the symmetric stretching vibrations of C–O bonds in –C–OH groups at 1130 cm^−1^ and the asymmetric stretching vibrations of C–O bonds in C–O–C groups at 1040 cm^−1^ could be observed for all of the prepared HBPEs. These indicate the occurrence of esterification. The averages of the molecular weights of the different HBPEs, which are measured by GPC and listed in Table 2, are 2160, 2660, 4150 and 5840 g/mol, respectively.

### 3.2. Viscosity

**Figure 3:**
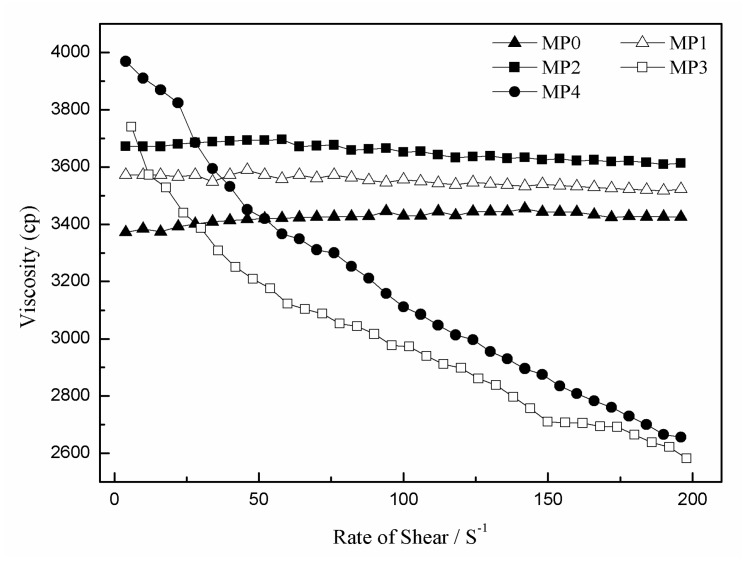
Shear viscosities as a function of shear rates for the PSf/HBPE/PEG400/DMAc casting solutions.

Figure 3 shows the viscosities of the casting solutions with different molecular weights of the HBPEs at 298 K. It was obvious that the initial viscosities of these casting solutions increased with the HBPE molecular weight. This phenomenon indicates that HBPE molecules act as nodes because of their hyperbranched structures in the casting solutions, which are beneficial to the entanglement of the PSf molecules and result in an increase in the initial viscosities. The higher the molecular weight of the HBPEs, the more the initial viscosities increased. Moreover, the shear-thinning phenomenon of these casting solutions became increasingly obvious. In particular, when the molecular weight of the HBPEs was above 4150 g/mol, the viscosities of MP3 and MP4 were lower than that of MP0. A possible explanation is that the number of PSf molecules tangled with higher-molecular-weight HBPE molecules is more than that of lower-molecular-weight HBPE molecules. By increasing the shear rate, the entanglement between the PSf molecules and the higher-molecular-weight HBPE molecules is easier to destroy than that of the lower-molecular-weight HBPE molecules. Consequently, the viscosity decreases. On the other hand, the globular HBPE molecules act as a lubricant and lead to the decrease of the casting solution viscosities. 

### 3.3. Light Transmittance

**Figure 4:**
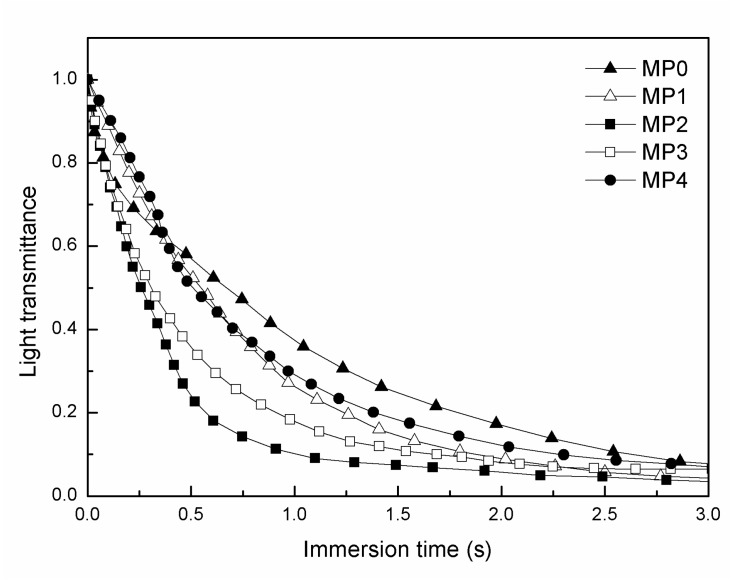
Light transmittance curves obtained by immersing the PSf dope solutions with different contents of the HBPEs into a pure water bath.

The light transmittance curves, as shown in Figure 4, revealed that the descending rate of the casting solution increased initially with the molecular weight of the HBPEs from 2160 to 2660 g/mol and then began to decrease when increasing the molecular weight of the HBPEs from 2660 to 5840 g/mol. This is because the HBPE molecule, which is an amphiphilic molecule, contains a hydrophilic shell and a hydrophobic core (as shown in Figure 1). When the molecular weight of the HBPEs was less than or equal to 2660 g/mol, the hydrophilic shell played a major role during the demixing process. This led to the casting solutions becoming less stable and increased the demixing rate. In contrast, when the molecular weight of the HBPEs was higher than 2660 g/mol, the amphiphilicity of the HBPE molecule played a predominant role during the demixing process. The HBPE molecules dissolved in both the poor solvent, PEG400, and the good solvent, DMAc, which improved the casting solution’s thermodynamic stability and decreased the demixing rate.

### 3.4. Morphology

**Figure 5:**
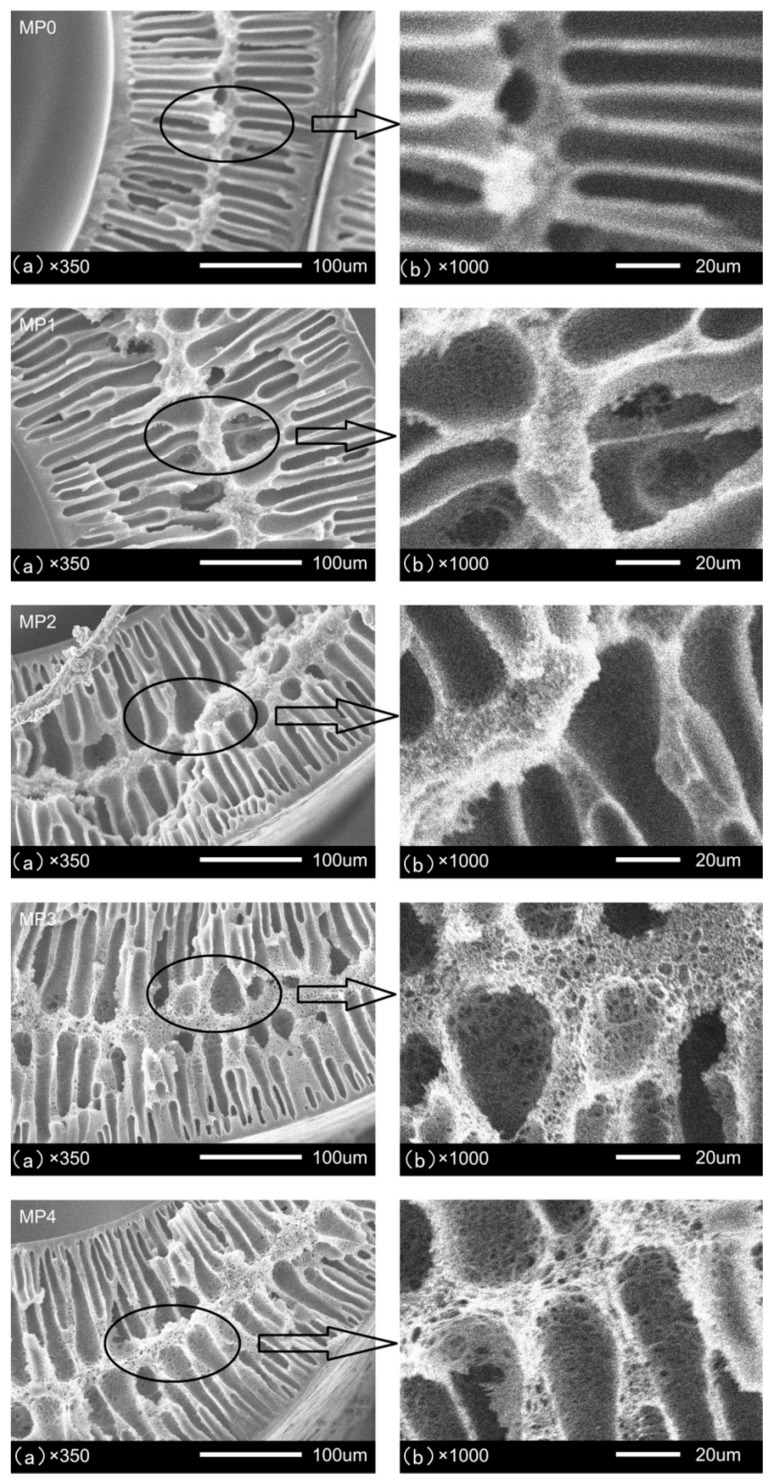
Scanning electron microscopy (SEM) micrographs of the PSf hollow fiber membranes; (**a**) cross-section; (**b**) enlarged cross-sections.

The molecular weight of the HBPEs has a significant effect on the morphologies of the PSf hollow fiber membranes, as shown in Figure 5. First, there was a double-skin layer and double-finger morphologies underneath the skin layers, which could be observed in all the prepared membranes. This indicates that instantaneous demixing was maintained in all of the formulations. Because the coagulation bath and the bore liquid are pure water, the instantaneous demixing happened on both the external and internal sides, which led to the double-finger-like structures and double-skin layers. Second, as the HBPE molecular weight increased from 2160 to 5840 g/mol, the skin layers thinned, and the inter-connected macroporous structure in the membrane became increasingly obvious. The explanation is that the casting solution takes on a relatively slow demixing rate, because the thermodynamic stability of the casting solutions improved, as shown in Figure 4. This is beneficial to the formation of the inter-connected macroporous structure, and this type of structure becomes increasingly distinct as the HBPE molecular weight increases. 

### 3.5. Hydrophilicity and Porosity

**Figure 6:**
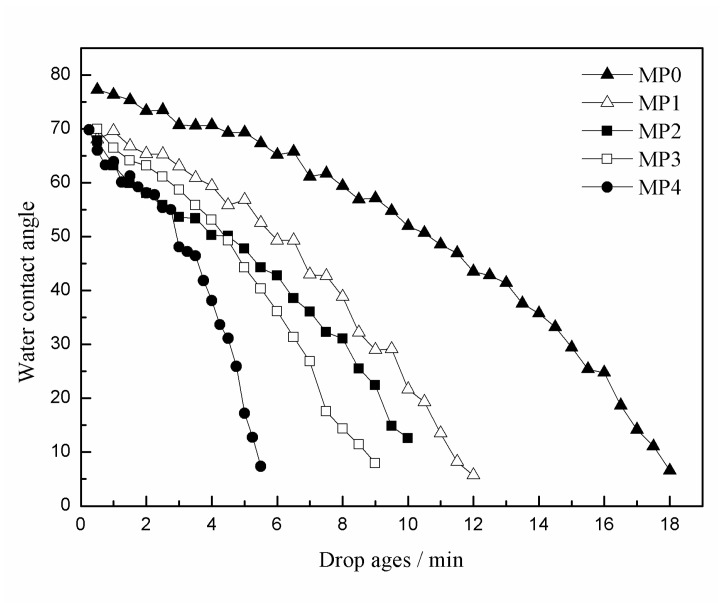
The dynamic pure water contact angles of the prepared PSf hollow fiber membranes.

The dynamic pure water contact angle (θ) is a firsthand method to characterize the hydrophilicity of the membrane, which is measured by continuous tracking. As shown in Figure 6, the starting pure water contact angle (θ_s_) of the pure PSf membrane MP0 was 77.3°, which was less than the 87.7° figure published by Ma et al. [35]. The reason is that a small amount of PEG400 is reserved in the membranes, which leads to a reduction in the pure water contact angle. With the addition of the HBPEs, the θ_s_ decreased from 77.3 to 68.1°, and the descending rate of the contact angle increased with the HBPE molecular weight. This is mainly because the periphery of the HBPE molecule has a hydrophilic shell, which leads to poor compatibility between the HBPEs and PSf. During the spinning process, the hydrophilic HBPE molecules migrated to the membrane surface. Therefore, the HBPE molecules on the membrane surface improved the hydrophilicity of the membranes and led to a decrease in the contact angle. With the increase in the molecular weight of HBPE, θ_s_ changed little, because the HBPE content was maintained at 1.0 wt % in all of the dope solutions.

**Figure 7:**
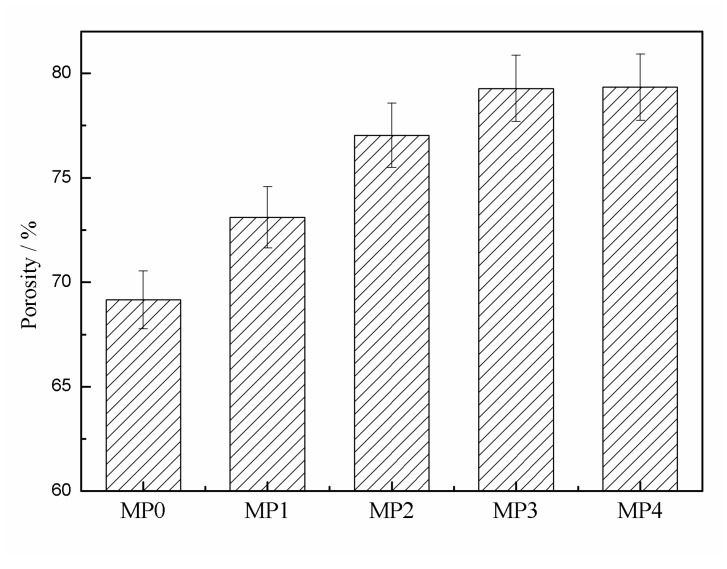
The porosity of the PSf hollow fiber membranes.

The porosities of the prepared PSf membranes with the different molecular weights of HBPEs are shown in Figure 7. The porosity significantly increased with the molecular weight of the HBPEs. The value of the porosity increased from 69.1% to 79.3%. These results validate the explanation of the SEM images in Figure 5. Meanwhile, the permeation rate of the pure water droplet increased with the increase in the porosity, which led to the decrease of the dope age.

### 3.6. Permeation Performance

**Figure 8:**
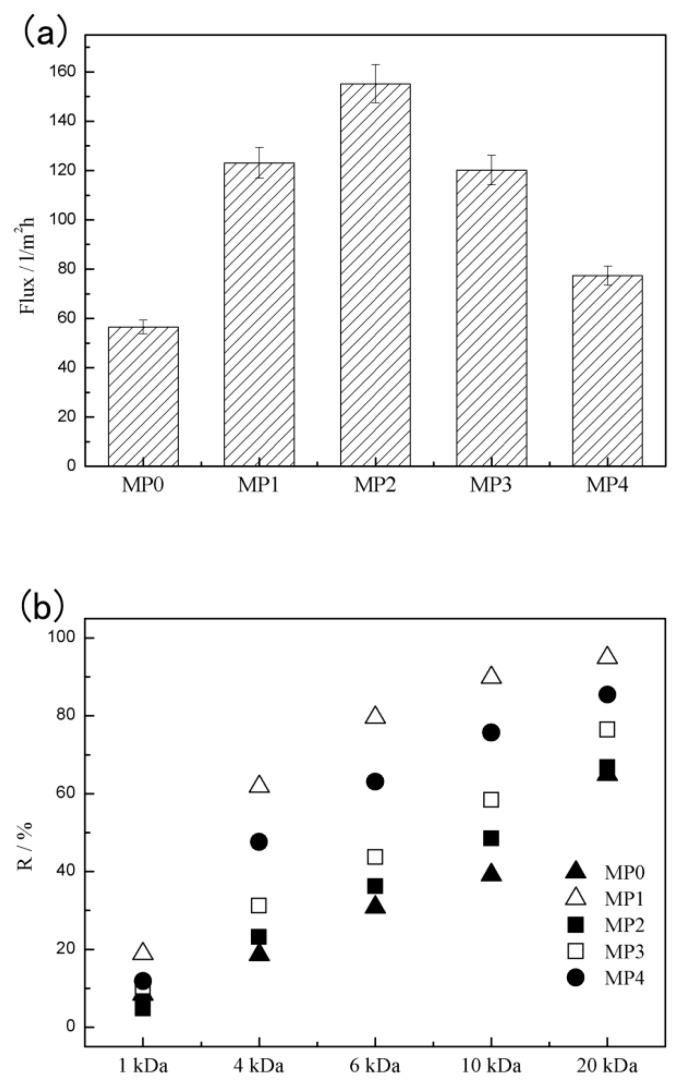
(**a**) Pure water permeation flux (*J_w_*) and (**b**) rejection rate (*R*) of the PSf hollow fiber membranes.

The pure water permeation flux (*J_w_*) and rejection rate (*R*) of the PSf hollow fiber membranes, which was measured by the dextran solute-transport experiments are shown in Figure 8. As shown in Figure 8a, the *J_w_* of the pure PSf membrane MP0 was 56.5 L/m^2^ h. With the addition of the HBPEs, the *J_w_* greatly increased to 123.1 L/m^2^ h (MP1). As the HBPE molecular weight increased continuously, the *J_w_* increased at the beginning and then decreased from 155.2 to 77.3 L/m^2^ h. As shown in Figure 8b, except for MP1, the R increased with the increasing of the HBPE molecular weight. It is well-known that membrane permeation performance depends largely on the membrane structure and hydrophilicity. When the HBPEs were introduced into the casting solutions, the hydrophilicity and porosity of the prepared PSf membranes (Figure 6 and Figure 7) increased with the HBPE molecular weight, which led to the flux improving. On the other hand, when the HBPE molecular weight further increased, the θ_s_ and porosity changed little (Figure 6 and Figure 7). Furthermore, when the HBPE molecular weight was higher than 2660 g/mol, the hydrophilic shell played a main role during the demixing process. This made a denser skin layer. Accordingly, the influences of dense skin layer gradually compensated for the effect of the increases in the hydrophilicity and porosity, which resulted in the decrease of flux and the increase of *R*.

### 3.7. Pore Size, Pore-Size Distribution and MWCO

**Figure 9:**
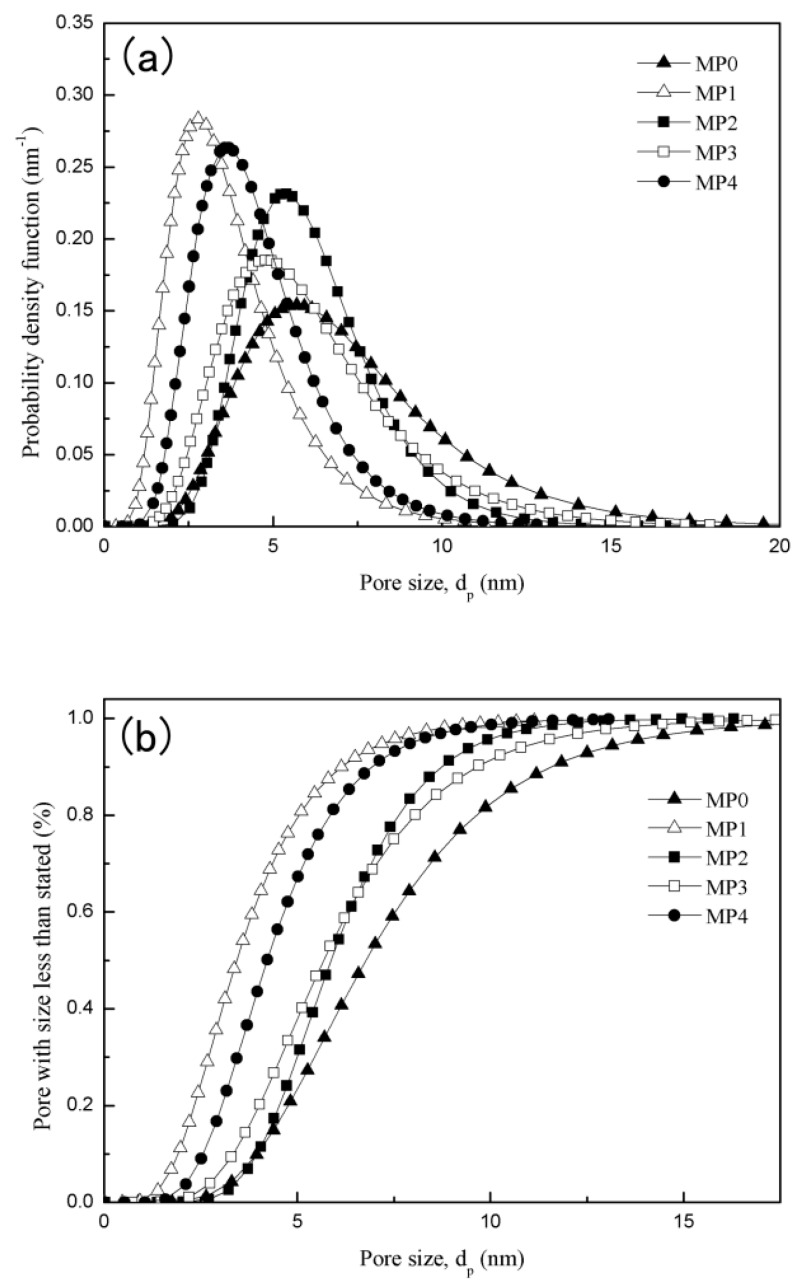
(**a**) The probability density function curves and (**b**) cumulative pore size distribution curves of various prepared PSf membranes.

The probability density function curves and cumulative pore-size distribution curves are presented in Figure 9a,b, respectively. The mean effective pore size and MWCO of various prepared membranes are listed in Table 3. As shown in Figure 5, the addition of HBPEs made the skin layer denser, which made ${\bar{d}}_{p}$ decrease from 6.78 to 3.43 nm. The reason may be that the hydrophilic hydroxyl of HBPEs leads to the formation of the dense skin layer. When the HBPE molecular weight increased from 2160 to 2660 g/mol due to the increase of porosity, ${\bar{d}}_{p}$ increased from 3.43 to 5.87 nm. Along with the increase of the HBPE molecular weight, the dense skin layer offset the influence of the increase of the porosity step by step, which decreased ${\bar{d}}_{p}$. With the addition of the HBPEs, the MWCO values decreased from 36,100 to 11,600 Da.

### 3.8. Mechanical Properties

**Figure 10:**
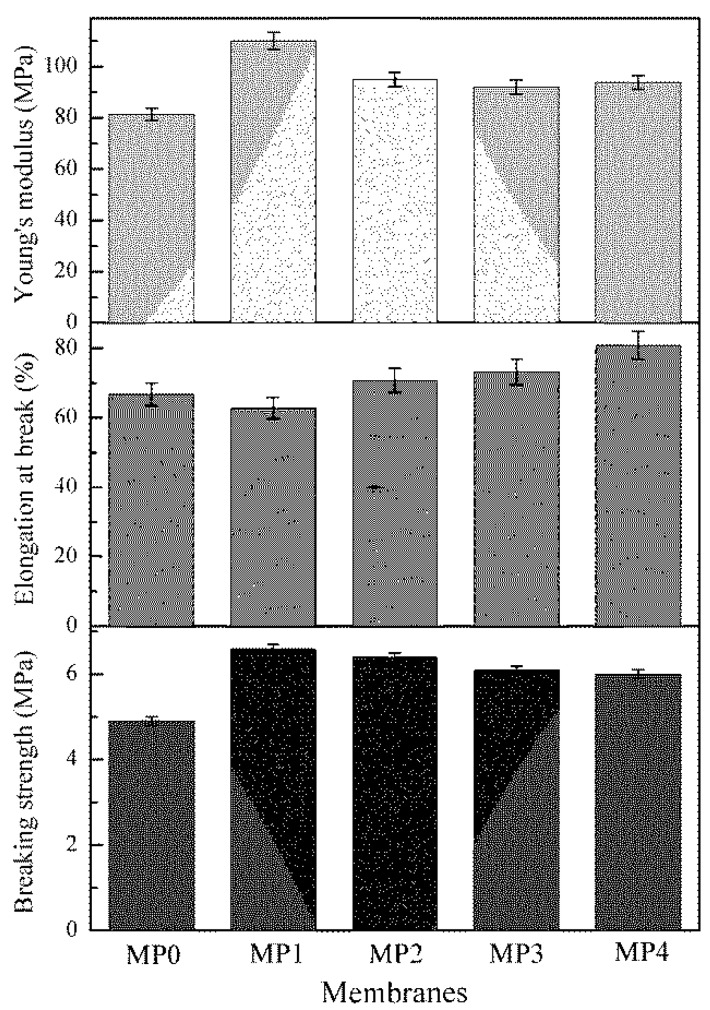
The mechanical properties of various prepared membranes.

The mechanical properties of the PSf membranes are shown in Figure 10. For the pure PSf hollow fiber membranes, the breaking strength was 4.9 MPa. As the HBPEs (SP-1) were added, the breaking strength increased to 6.6 MPa, which was due to the entanglement between the PSf molecules and HBPEs. When the molecular weight increased from 2160 to 5840 g/mol, the breaking strength gradually decreased to 6.1 MPa. The results correlate to those of the SEM images (the inter-connected macroporous structure became increasingly obvious, as shown in Figure 5) of the prepared PSf hollow fiber membranes. 

As to the elongation at break, with the addition of the HBPEs, the elongation decreased to 62.7% initially, and then the elongation increased with the HBPE molecular weight. The Young’s modulus of the PSf membranes with the HBPEs (MP1, MP2, MP3 and MP4) was larger than that of the pure PSf membranes (MP0). This indicates that the HBPEs can effectively improve the membrane stiffness.

A comparison of the mechanical properties of the PSf hollow fiber membranes fabricated in this study with PSf membranes in other published papers is shown in Table 4. The high breaking strength and Young’s modulus could be obtained in this work when the molecular weight of the HBPEs was 2160 g/mol. Moreover, it can be seen that some reported membranes based on lignocelluloses (LGC) and caramel displayed better breaking strength. Therefore, the addition of the HBPEs is beneficial to the improvement of the mechanical properties of the membrane.

## 4. Conclusions

Four different HBPEs based on PER and bis-MPA, which contained a hydrophilic shell and a hydrophobic core, were successfully synthesized by esterification reaction. The molecular weights of the prepared HBPEs were 2160, 2660, 4150 and 5840 g/mol, respectively. These HBPEs were first used as additives to modify the PSf membranes. With the addition of the HBPEs, the initial viscosities of the casting solutions increased, and the shear-thinning phenomenon became increasingly obvious. During the spinning process, the membrane formation mechanism belonged to instantaneous demixing. Due to the addition of the HBPEs, the prepared PSf membranes presented a co-existing morphology of double finger-like and sponge-like structures. Furthermore, the hydrophilicity of the membranes improved obviously, and the breaking strength and Young’s modulus increased with the decrease of the molecular weight of the HBPEs. These phenomena were significantly different from the linear additives.

## Figures and Tables

**Table 1:** Compositions of polysulfone (PSf)/(poly)ethyleneglycol (PEG400)/dimethylacetamide (DMAc)/hyperbranched polyester (HBPE) casting solutions.

Membrane Number	Casting Solution Compositions (wt %)				HBPE Code Name
	PSf	HBPE	PEG400	DMAc	
**MP0**	18.0	0	18.7	62.3	
MP1	18.0	1.0	18.7	62.3	SP-1
MP2	18.0	1.0	18.7	62.3	SP-2
MP3	18.0	1.0	18.7	62.3	SP-3
MP4	18.0	1.0	18.7	62.3	SP-4

**Table 2:** The gel permeation chromatography (GPC) results of the prepared HBPEs.

	${\bar{M}}_{w}$	${\bar{M}}_{n}$	${\bar{M}}_{w}/{\bar{M}}_{n}$
SP-1	2160	1910	1.13
SP-2	2660	2370	1.12
SP-3	4150	3490	1.19
SP-4	5840	4570	1.27

**Table 3:** The mean effective pore size and molecular weight cut-off of various prepared membranes.

Membranes	${\bar{d}}_{p}$ (nm)	MWCO (Da)
MP0	6.78	36,100
MP1	3.43	11,600
MP2	5.87	21,700
MP3	5.68	26,100
MP4	4.22	14,400

**Table 4:** Comparison of mechanical properties with other studies.

Membrane	Preparation Method	Filler Loading	Breaking Strength (MPa)	Elongation at Break (%)	Young’s Modulus (MPa)	Ref.
PSf	NIPS	1.0 wt %HBPEs-PER (${\bar{M}}_{w}$ = 2160)	6.6	62.7	110.3	This work
PSf	NIPS	1.0 wt %HBPEs-PER (${\bar{M}}_{w}$ = 11,200)	6.2	73.5	107.0	[23]
PSf	NIPS	1.0 wt %HBPEs-TMP (${\bar{M}}_{w}$ = 2470)	6.1	84.4	89.1	[25]
PSf	Heat treatment	Tension heating for 1 h at 185 °C	7.31	-	-	[36]
PSf	Heat treatment	Tension heating for 1 h at 195 °C	5.51	-	-	[36]
PSf	DIPS	-	5.5	6.9	-	[37]
PSf	DIPS	1.0 wt % LGC	8.7	10.3	-	[37]
PSf	DIPS	1.0 wt % LGC and 0.75 wt % caramel	10.8	13.4	-	[37]
